# Comparative Evaluation of Osseointegrated Dental Implants Based on Platform-Switching Concept: Influence of Diameter, Length, Thread Shape, and In-Bone Positioning Depth on Stress-Based Performance

**DOI:** 10.1155/2013/250929

**Published:** 2013-06-19

**Authors:** Giuseppe Vairo, Gianpaolo Sannino

**Affiliations:** ^1^Department of Civil Engineering and Computer Science, University of Rome “Tor Vergata,” Via del Politecnico 1, 00133 Rome, Italy; ^2^Department of Oral Health, University of Rome “Tor Vergata,” Viale Oxford, 00133 Rome, Italy

## Abstract

This study aimed to investigate the influence of implant design (in terms of diameter, length, and thread shape), in-bone positioning depth, and bone posthealing crestal morphology on load transfer mechanisms of osseointegrated dental implants based on platform-switching concept. In order to perform an effective multiparametric comparative analysis, 11 implants different in dimensions and in thread features were analyzed by a linearly elastic 3-dimensional finite element approach, under a static load. Implant models were integrated with the detailed model of a maxillary premolar bone segment. Different implant in-bone positioning levels were modeled, considering also different posthealing crestal bone morphologies. Bone overloading risk was quantified by introducing proper local stress measures, highlighting that implant diameter is a more effective design parameter than the implant length, as well as that thread shape and thread details can significantly affect stresses at peri-implant bone, especially for short implants. Numerical simulations revealed that the optimal in-bone positioning depth results from the balance of 2 counteracting effects: cratering phenomena and bone apposition induced by platform-switching configuration. Proposed results contribute to identify the mutual influence of a number of factors affecting the bone-implant loading transfer mechanisms, furnishing useful insights and indications for choosing and/or designing threaded osseointegrated implants.

## 1. Introduction

In the last three decades and in the field of the prosthetic dentistry, features of dental implants and surgical procedures have been developed and enhanced aiming to ensure predictable results and to improve function and aesthetics in completely or partially edentulous patients [1].

A dental implant is a biocompatible device, surgically placed into mandibular or maxillary bone for supporting a prosthetic tooth crown, and thus allowing the replace of the teeth lost due to caries, periodontal disease, injuries, or other reasons. Worldwide statistics show that a high success rate of dental implants (over 95%) occurs if implants are properly designed and manufactured, and if they are inserted in a bone segment characterized by good quality and quantity (e.g., [2–4]). Nevertheless, success of the prosthetic treatment is widely affected by a number of factors that can change the biomechanichal coupling between implant and bone, such as implant location, mechanical and morphological properties of bone, mechanical and geometrical features of implant, and type and magnitude of the load transferred by the implant to the bone, as well as by host factors such as smoking and bacterial environment [5–7].

A crucial aspect that determines the effectiveness of a dental implantation is identified by the proper development of the osseointegration process at the bone-implant interface. This process is similar to the healing process in bone fracture [7–9] and arises from remodeling mechanisms that involve a number of cellular and extra­cellular coupled biomechanical features. After the implantation, the gap between the implant and the host bone is rapidly filled by blood clots that are afterwards substituted by a trabecular network. The latter generally evolves towards the formation of lamellar bone that, in turn, undergoes a maturation process that modifies density and mechanical properties of the tissue [8–11]. At the end of the healing process, the mature bone is directly in contact with the implant surface, leading to an interfacial binding that allows to enhance loading transfer mechanisms from prosthetic crown to the bone [12, 13].

Nevertheless, a proper osseointegration process may be counteracted by the activation of histological resorption mechanisms [9, 14–16] that can induce bone weakening or loss at the peri-implant region. Bone resorption mainly affects the bone region around the implant neck, producing a cratering morphology, and it may be activated by surgical trauma or bacterial infection, as well as by overloading states [4, 5, 14–22]. Under functional or pathological (e.g., induced by bruxism) loads, overloading at the peri-implant bone may occur by a shortcoming in load transfer mechanisms, mainly due to bad occlusion, improper implant use, wrong prosthesis and/or implant design, and improper implant placement. In these cases, high stress concentrations are induced at the bone-implant interfaces, leading to possible physiologically inadmissible strains that activate bone resorption [23, 24]. Clinical trials and follow-up analyses [2–4, 17, 18] have shown that the implant failure may generally occur if the bone resorption process significantly evolves from a crestal initiation. Depending on implant features, positioning and loads, this process may become instable, leading to a progressive increase in stress intensity at the peri-implant interface [19] that, in turn, further contributes to the progressive overload-induced bone loss.

Recent clinical evidence [25–29] suggests that cratering phenomena may be significantly limited when the connection diameter of the abutment is narrower than the implant collar and when an implant subcrestal positioning is applied. In this case, probably due to the different position of the implant/abutment microgap and to the different stress pattern induced at the peri-implant regions with respect to a crestal positioning, remodeling process generally evolves allowing bone apposition on the horizontal implant surface and thus transferring the biological width from the vertical to the horizontal level (platform switching) [30–34].

In order to improve durability and clinical effectiveness of rehabilitations based on such an approach, mechanical and biological factors mainly affecting loading transfer from implant to bone have to be properly identified and quantified. Thereby, optimized implant designing strategies and surgical protocols could be traced, allowing us to minimize overloading risks and marginal bone loss, as well as contributing to ensure predictable clinical results. 

In the recent specialized literature many authors have proposed results based on well-established in vivo, in vitro, and in silico approaches, aiming to investigate main biomechanical factors influencing the preservation of the peri-implant marginal bone as well as the stress/strain patterns induced by osseointegrated implants [4, 26–29, 35, 36]. In this context, finite-element method has been widely used in the last years to analyze the influence of implant and prosthesis design [37–40], of magnitude and direction of loads [41–44], and of bone mechanical properties [45–47], as well as for modeling different clinical scenarios [48–54]. Nevertheless, many effects related to the implant design and to the in-bone positioning depth, as well as their mutual influence on the stress-based implant performance, have not yet been completely understood and clarified, especially for implants based on platform-switching concept.

In this study, 11 threaded dental implants, based on platform-switching concept and different for dimensions and thread type, were compared via a multiparametric three-dimensional (3D) finite-element approach. Accurate and convergent bone-implant models, defined by considering a maxillary premolar bone segment, have been solved by employing a linearly elastic displacement-based formulation and considering a static functional loading condition. Stress distributions were numerically evaluated at the peri-implant regions on both compact and cancellous bone, furnishing quantitative risk measures of bone physiological failure. Proposed numerical results highlighted the influence of implant shape, in terms of implant length and diameter as well as in terms of thread features, on possible overloading risks and on mechanisms of load transfer. The influence of implant positioning in bone was also investigated by considering numerical models based on both crestal and subcrestal implant placements. Finally, in the case of a crestal positioning and in order to contribute to the understanding of the biomechanical relationship between mechanical stimuli and marginal bone loss, several numerical simulations were carried out for analyzing the effects of different cratering levels on stress patterns at the peri-implant bone.

## 2. Material and Methods

**Figure 1:**
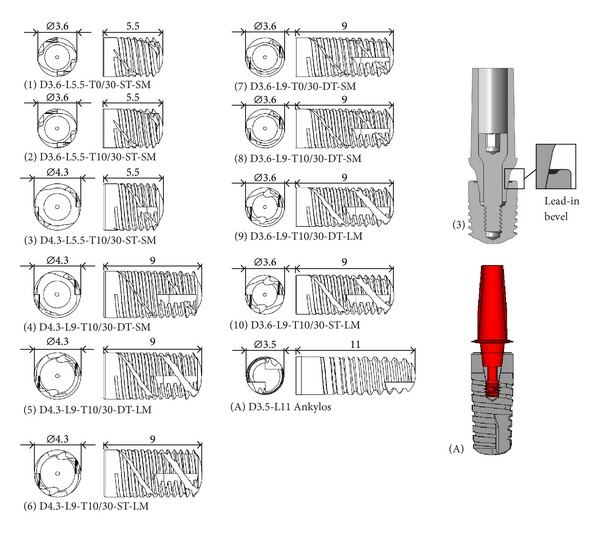
Threaded dental implants analyzed in this study. Notation and examples of implant-abutment coupled systems that allow a platform-switching configuration.

Ten threaded dental implants, different in diameter (*D*), length (*L*), thread shape, and geometrical concept, were analyzed and compared with each other and with an Ankylos implant (Dentsply Friadent, Mannheim, Germany) characterized by *D* = 3.5 mm and *L* = 11.0 mm.  Figure 1 summarizes the main geometrical features of the implants analyzed in this study, introducing also the corresponding notation. Symbols T0/30 and T10/30 refer to the implant thread: T0/30 denotes a saw-tooth thread with the side angled at 120° with respect to the implant axis and with a free thickness of 0.33 mm at the internal diameter; T10/30 denotes a trapezoid-shaped thread with sides angled at 120° and 100° with respect to the implant axis and with a free thickness of 0.25 mm at the internal diameter. Both threads are characterized by two starts with a conical helix having the same anomaly and with an effective pitch of 1.2 mm. Moreover, symbol ST indicates that both starts exhibit the same thread truncation, resulting in a maximum thread depth of 0.38 mm, whereas symbol DT denotes implants with a different thread truncation for each start, resulting in maximum thread depths of 0.19 mm and 0.38 mm, respectively. Implants, except the Ankylos device, have also a helical milling, with the effective pitch equal to the implant threaded length. Depending on width and depth of cut, small and large millings are identified by symbols SM and LM, respectively. Implants denoted by 1 to 10 in Figure 1 were characterized by an internal lead-in bevel extending from the outer most diameter of the implant platform into a flattened area or ledge. Moreover, implants analyzed in this study have vertical cutting grooves for self-tapping insertion and have been coupled with abutments characterized by connection diameters narrower than the implant collars, thereby allowing a platform-switching configuration (see Figure 1).

**Figure 2:**
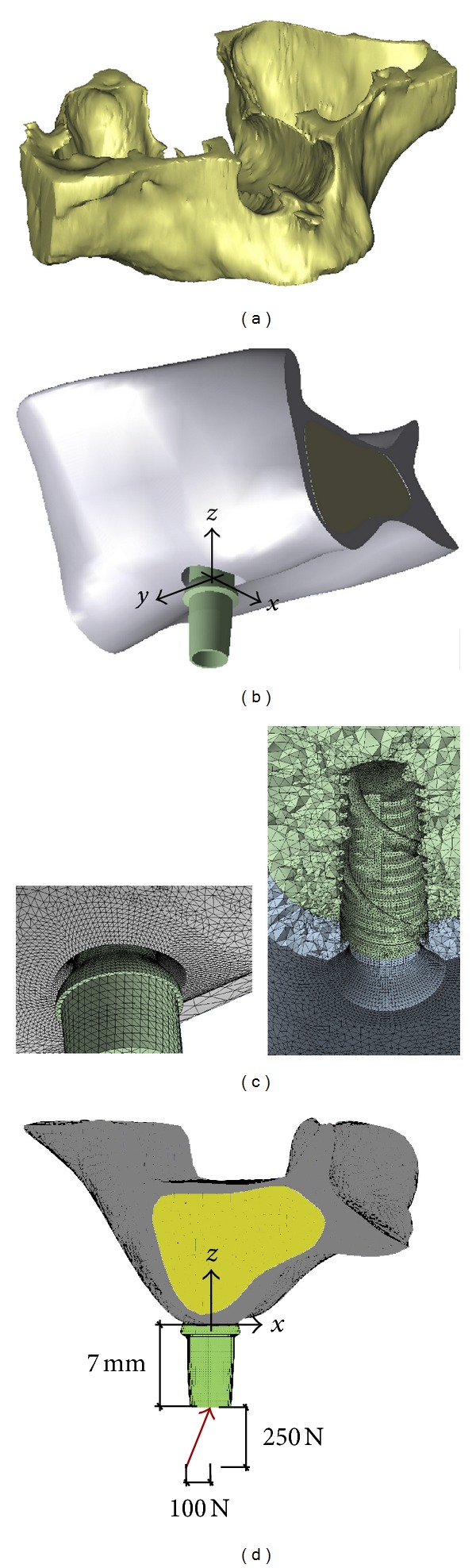
(a) Three-dimensional solid model of the edentulous maxilla considered in this study and obtained by a segmentation process based on multislice computed tomography (MSCT). (b) Submodel of the second premolar maxillary region, defined by considering two coronal sections at the distance of 40 mm along the mesiodistal direction (*y* axis) and positioning implants at the mid-span of the bone segment. (c) Examples of mesh details. (d) Loading condition.

Models of implants and abutments were built up by using a parametric CAD software (SolidWorks 9; Dessault Systèmes, Concord, Mass) and, in order to perform consistent comparisons, they were integrated within the model of a premolar bone segment, obtained by the three-dimensional (3D) model of an edentulous maxilla (Figure 2). The latter was reconstructed starting from multislice computed tomography (MSCT) scans and by using a modeling commercial software (Mimics, Materialise HQ, Leuven, Belgium). Moving from the different hues of gray displayed in the planar CT scans, corresponding to different radiolucency levels of substances with different density values, the software allowed us to distinguish between mineralized and soft tissues, by filtering pixels with a suitable Hounsfield units (HU) [55]. In detail, disregarding gingival soft tissues, the solid model of the maxillary jaw was obtained by a segmentation procedure of voxels identified by HU > 150 (Figure 2(a)) and based on a home-made smoothed linear interpolation algorithm. Cortical and trabecular regions were distinguished, considering 150 < HU ≤ 750 for the cancellous bone and HU > 750 for the cortical bone. With the aim of improving the model quality, ad hoc local geometry adjustments were performed, ensuring that the cortical bone regions were characterized by a mean thickness of about 2 mm. Starting from the complete maxillary jaw model, the finite-element computations were carried out on a submodel of the second premolar region, defined by considering two coronal sections at the distance of 40 mm along the mesiodistal direction (*y*, in Figure 2(b)) and positioning implants at the mid-span of the bone segment.

**Figure 3:**
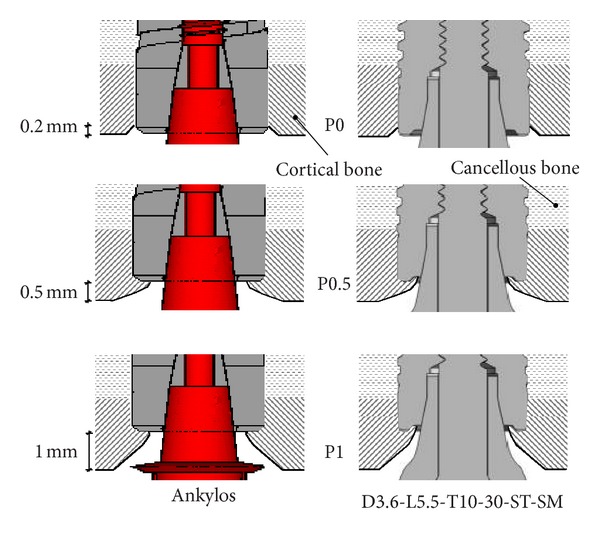
Modeling of crestal bone geometries and different configurations of implant in-bone positioning analyzed in this study. In the case of the configuration P0, a crestal bone loss of about 10% in thickness is depicted.

A subcrestal positioning was firstly investigated, by considering implant models positioned with the crestal platform at 1 mm depth with respect to the outer bone surface. As a notation rule, in the foregoing this configuration will be denoted as P1. Moreover, in order to analyze the positioning influence for implants similar in diameter and length, numerical models relevant to the implants D3.6-L9-T10/30-DT-SM and Ankylos (indicated as 8 and A, resp., in Figure 1) were analyzed by considering a crestal positioning (i.e., with the implant platform at the level of the outer bone surface and denoted as P0); an intermediate subcrestal positioning at 0.5 mm depth (denoted as P05). With the aim of reproducing as realistically as possible the physiological structure of the compact bone arising around a functioning implant after a healing period, different crestal geometries were modeled. In particular, in agreement with well-established clinical evidence [25–27] and modeling approaches [40, 47, 53], and as sketched in Figure 3, a crestal bone apposition at the implant platform of about 0.25 mm in mean thickness was modeled for subcrestal placements (i.e., for models denoted as P1 and P05), whereas a marginal bone loss of 10% in cortical thickness was modeled for the crestal positioning (P0). For implants 8 and A crestally placed (P0), the influence of different levels of marginal bone loss (0–50% in cortical thickness) was also analyzed.

All the involved materials were modeled as linearly elastic with an isotropic constitutive symmetry, and all material volumes were modeled as homogeneous. Thereby, bone living tissue was described by considering a dry-material model, wherein viscous and fluid-solid interaction effects were neglected. Implants and abutments were assumed to be constituted by a titanium alloy, Ti6Al4V, whose Young's modulus and Poisson's ratio were 114.0 GPa and 0.34, respectively [56]. Bone elastic properties were assumed to approximate type II bone quality [57] and, in agreement with data available in the literature [40, 47, 58], they were set as follows:

- Poisson's ratio of the bone tissue (both cortical and trabecular) equal to 0.30;
- Young's modulus of the cortical bone equal to 13.7 GPa;
- Young's modulus of the cancellous bone equal to 0.5 GPa, corresponding to a mean bone density of about 0.5 g·cm^−3^ [59].

Finite-element simulations were carried out considering a static load applied at the top of the abutments without any eccentricity with respect to the implant axis and angled with respect to the occlusal plane of about 68°. The lateral force component along the buccolingual direction (*x*, in Figure 2) was assumed to be equal to 100 N and the vertical intrusive one (along *z*, in Figure 2) was 250 N. In order to allow consistent comparisons, abutments were adjusted in such a way that the application points of the load were 7 mm from the bone insertion surface in all numerical models (see Figure 2(d)).

Complete osseous integration between implant and bone tissue was assumed, enforcing the continuity of the displacement field at the bone-implant interface. Furthermore, displacement continuity is imposed between each component of a given prosthetic device. As regards boundary conditions for numerical models describing the coupled bone-implant system, all displacement degrees of freedom were prevented for any boundary node lying on the coronal sections delimiting the bone submodel. In agreement with the theory of elasticity [60], since the distance between submodel boundary sections and the implant location was much greater than the implant's characteristic dimensions, these boundary conditions did not significantly affect stress-based comparative results at the peri-implant regions.

Discrete finite-element meshes were generated by employing elements based on a pure displacement formulation and were analyzed with a commercial solver code (Ansys 13.0; Ansys Inc., Canonsburg, PA). Computational models were obtained by considering 10-node tetrahedral elements [61], with quadratic shape functions and three degrees of freedom per node. In order to ensure suitable accuracy of the numerical finite-element solutions at the peri-implant regions, mesh-size for the bone-implant models was set up as a result of a convergence analysis, based on the coupled estimate within the multiregion computational domain of the displacement error norm and of the energy error norm [61]. In detail, following the numerical procedure proposed by Zienkiewicz and Zhu [62], implemented in the Ansys environment and recently applied for prosthetic dental applications [47], the proposed numerical results were obtained by solving discrete models based on *h*_0_/*D* = 0.1 and *h*_*i*_/*D* = 0.01, *h*_0_ and *h*_*i*_ being mean mesh-size away from the bone-implant interface and close to the peri-implant regions, respectively. This choice was proved to ensure a good numerical accuracy, resulting for all models analyzed in this study in a value of the energy error norm lower than 5% and in a value of the displacement error norm lower than 0.5%.

Jaw submodel treated by a single-implant prosthesis was numerically compared by analyzing stress distributions arising at the peri-implant regions. The Von Mises equivalent stress (*σ*_VM_), often used in well-established numerical dental studies (e.g., [35–54, 63, 64]), was used as a global stress indicator for characterizing load transfer mechanisms of a given implant. Nevertheless, the Von Mises stress measure, always positive in sign, does not allow a distinction between tensile and compressive local stresses. Since experimental evidence [24, 58, 65] confirms that bone physiological failure and overload-induced resorption process are differently activated in traction and compression, more effective and direct risk indications were obtained by analyzing stress measures based on principal stresses (*σ*_*i*_, with *i* = 1,2, 3) [44, 47, 53, 63, 64]. In detail, in a given material point *P* of the computational domain that models the peri-implant bone, the following stress measures were computed:

(1) $$\begin{array}{c} \sigma_{C}\left(P\right)=\min\left\{\sigma_{1}\left(P\right),\sigma_{2}\left(P\right),\sigma_{3}\left(P\right),0\right\},\\ \sigma_{T}\left(P\right)=\max\left\{\sigma_{1}\left(P\right),\sigma_{2}\left(P\right),\sigma_{3}\left(P\right),0\right\}, \end{array}$$

*σ*_*C*_ and *σ*_*T*_ having the meaning of maximum compressive and maximum tensile stress in *P*, respectively. Therefore, in order to combine effects induced on bone by compressive and tensile local states which are simultaneously present, the bone safety in *P* against overloading-related failure/resorption process activation was postulated to occur if the following inequality was satisfied:

(2) $$\begin{array}{c} R=\frac{\left|\sigma_{C}\right|}{\sigma_{C0}}+\frac{\sigma_{T}}{\sigma_{T0}}\leq 1, \end{array}$$

where symbol |*a*| denotes the absolute value of the scalar quantity *a* and where *σ*_*T*0_, *σ*_*C*0_ are the admissible stress levels in pure traction and compression, respectively. Accordingly, the dimensionless positive quantity *R* can be thought of as a quantitative risk indicator, such that the condition *R* > 1 identifies a local critical state of bone with respect to overloading effects. By assuming that overloads occur when ultimate bone strength is reached, in this study it was assumed that *σ*_*T*0_ = 180 MPa and *σ*_*C*0_ = 115 MPa for cortical bone and *σ*_*T*0_ = *σ*_*C*0_ = 5 MPa for trabecular bone [58, 65].

**Figure 4:**
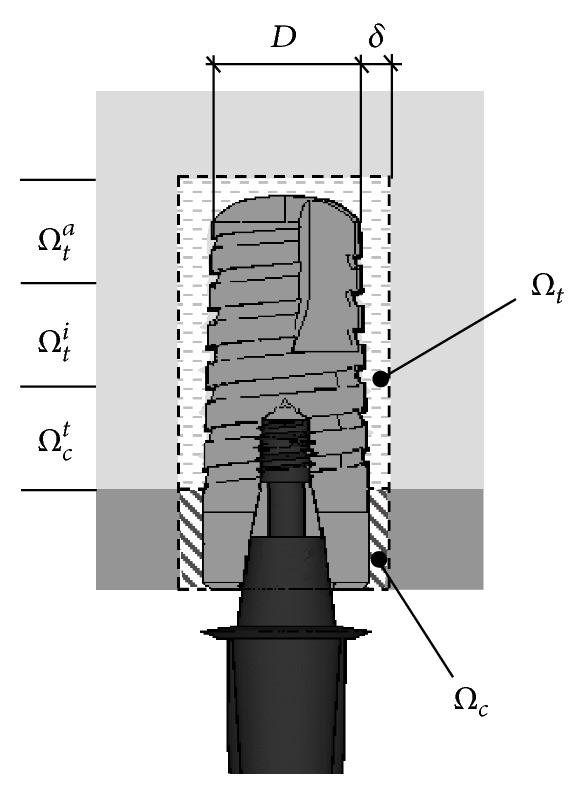
Control regions employed for computing the local stress measures and the overloading risk index *R* at the bone-implant interface.

In order to perform significant numerical comparisons, the previously introduced stress measures and the risk index *R* were computed for each implant within a control volume *Ω*, defined by considering a bone layer surrounding the implant with a mean thickness *δ*. With reference to the sketch in Figure 4, the region *Ω* has been conveniently considered as subdivided in its complementary parts *Ω*_*c*_ and *Ω*_*t*_ (such that *Ω* = *Ω*_*c*_ ∪ *Ω*_*t*_), representing cortical and trabecular control regions, respectively. In turn, *Ω*_*t*_ has been further subdivided, by 2 planes orthogonal to the implant axis, into 3 complementary control subregions having equal length along the implant axis. These three trabecular regions will be denoted as *Ω*_*t*_^*c*^ (crestal region), *Ω*_*t*_^*i*^ (intermediate region), and *Ω*_*t*_^*a*^ (apex region). Results discussed in the foregoing were obtained by assuming *δ*/*D* = 0.25, and they refer to average and peak values of *σ*_VM_, *σ*_*C*_, *σ*_*T*_, and *R* over *Ω*_*c*_, *Ω*_*t*_^*c*^, *Ω*_*t*_^*i*^,  *Ω*_*t*_^*a*^. These results were computed via a postprocessing phase carried out by means of a MatLab (The MathWorks, Inc., Natick, MA) home-made procedure, taking as input by the solver code some primary geometrical and topological data (nodes and elements lying in *Ω*), as well as stress solutions at the finite-element Gauss points within *Ω*.

## 3. Results

### 3.1. Subcrestal Positioning P1

**Figure 5:**
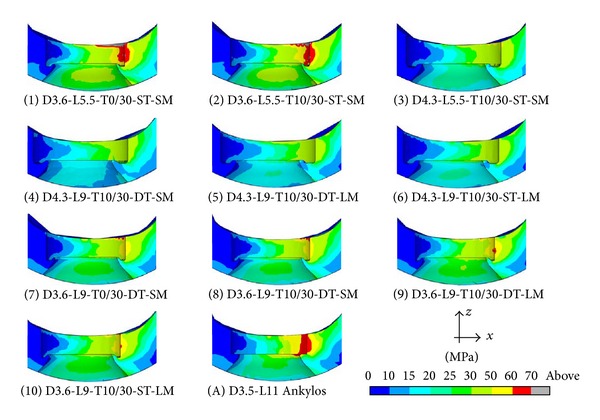
Von Mises stress contours (blue: 0; red: 70 MPa) at the coronal section *y* = 0 for implants defined in Figure 1 and in the case of the subcrestal positioning P1 (see Figure 3). Cortical peri-implant bone interface.

**Figure 6:**
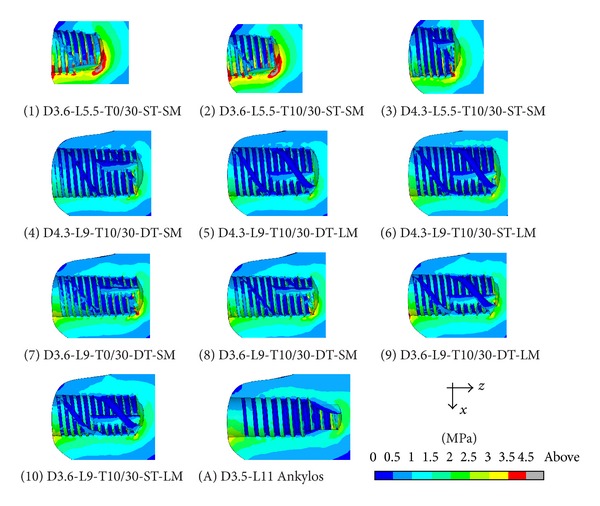
Von Mises stress contours (blue: 0; red: 4.5 MPa) at the coronal section *y* = 0 for implants defined in Figure 1 and in the case of the subcrestal positioning P1 (see Figure 3). Trabecular peri-implant bone interface.

**Figure 7:**
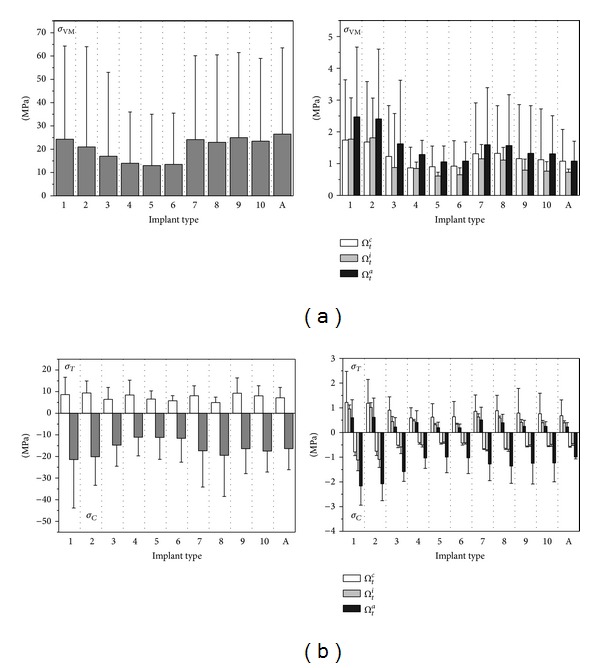
Von Mises ((a), *σ*_VM_) and principal ((b), *σ*_*T*_ tensile and *σ*_*C*_ compressive) stress measures at cortical (left side) and trabecular (right side) bone-implant interface for implants defined in Figure 1 and in the case of the subcrestal positioning P1 (see Figure 3). Average (bars) and peak (lines) values.

**Figure 8:**
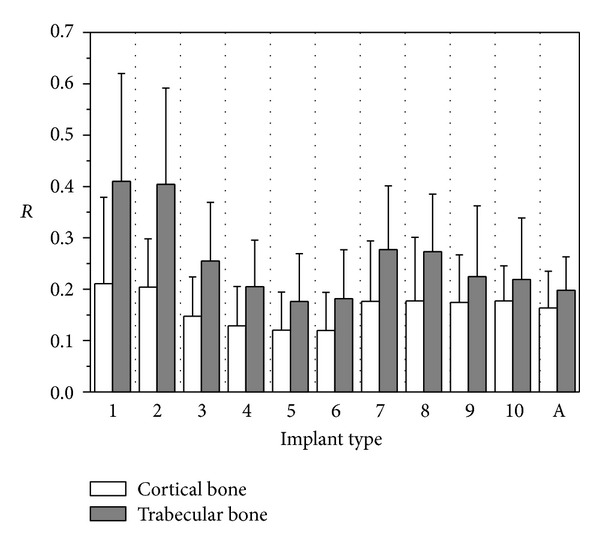
Overloading risk index *R* computed at cortical and trabecular peri-implant bone for implants defined in Figure 1 and in the case of the subcrestal positioning P1 (see Figure 3). Average (bars) and peak (lines) values.

For implants introduced in Figure 1 and considering the subcrestal positioning P1 (see Figure 3), Figures 5 and 6 show Von Mises stress distributions relevant to the loading coronal plane *y* = 0, computed via the present 3D finite-element approach at the peri-implant cortical and trabecular bone regions. Moreover, Figure 7 shows average and peak values over the control volumes *Ω*_*c*_ and *Ω*_*t*_ (see Figure 4) of *σ*_VM_ and of the principal stress measures defined by (1). Finally, Figure 8 highlights mean and peak values of the overloading risk index *R* computed at both trabecular and cortical peri-implant bone regions.

By assuming complete osseous integration, the highest stress concentrations were computed at the cortical bone near the implant neck. There, stress patterns were significantly affected by implant diameter (*D*) and bone-implant interface length (*L*). In detail, by increasing *D* and/or by increasing *L* mean and peak stress values decreased in *Ω*_*c*_ and *Ω*_*t*_, and stress distributions tended to be more homogenous. Compressive mean and peak values at the cortical peri-implant region always prevailed with respect to the corresponding tensile states. This occurrence was not generally respected at the trabecular interface, wherein tensile stresses were higher at the crestal region (*Ω*_*t*_^*c*^) and smaller at the implant apex (*Ω*_*t*_^*a*^) than the compressive stresses. Nevertheless, the highest trabecular stress peaks were associated with the compressive states arising in *Ω*_*t*_^*a*^ (see Figure 7(b)).

Referring to the notation introduced in Figure 1, implants denoted by D4.3-L9 (i.e., labeled as 4, 5, and 6) exhibited the best stress performances, resulting in the smallest values of the stress measures as well as in the smallest values of the overloading risk index *R*. On the contrary, implants denoted by D3.6-L5.5 (labeled as 1 and 2) numerically experienced the worst loading transmission mechanisms. Moreover, the stress-based performance of the commercial implant Ankylos D3.5-L11 was estimated as fully comparable with that of the threaded implants D3.6-L9 (labeled as 7, 8, 9, and 10), although the greater Ankylos' length induced more favorable stress distributions at the trabecular bone, especially referring to the compressive states arising at the implant apex (see Figure 7(b)).

Proposed results clearly show that the parameter that mainly affects the implant stress-based performances is the diameter *D*, irrespective of the length *L*. In fact, by comparing stress results relevant to implant 2 with those of implant 3, that is, by increasing *D* of about 20% (passing from *D* = 3.6 mm to *D* = 4.3 mm) when *L* = 5.5 mm, compressive (resp., tensile) peak values reduced of about 27% in both *Ω*_*c*_ and *Ω*_*t*_ (resp., 20% in *Ω*_*c*_ and 30% in *Ω*_*t*_). On the contrary, by comparing stress results relevant to implant 2 with those of implant 9, that is, by increasing *L* of about 60% (passing from *L* = 5.5 mm to *L* = 9 mm) when *D* = 3.6 mm, compressive peaks reduced only by about 16% (resp., 26%) at the cortical (resp., trabecular) bone, whereas tensile peaks were almost comparable. These considerations are qualitatively applicable also when the overloading risk index *R* is addressed (see Figure 8), leading to similar conclusions.

Within the limitations of this study, overloading risks were greater in cancellous region than those in cortical, and proposed numerical results highlighted that, under the simulated loading condition, the safety inequality *R* < 1 was everywhere satisfied in bone for all the analyzed implants.

Moreover, the proposed numerical results suggest that thread shape and thread details can induce significant effects on local stress patterns in bone around implants. In particular, the use of the same thread truncation (ST) for both thread starts induced a more uniform local stress distributions than the case characterized by a different thread truncation (DT), since all the threads had practically the same engaged depth. As a result, mean and peak values of *σ*_*T*_ reduced at the cortical bone passing from DT to ST, as it is shown in Figure 7(b) by comparing results relevant to implants 5 and 6 (peaks reduced of about 20% and mean values of about 13%) and to implants 9 and 10 (peaks reduced of about 23% and mean values of about 18%).

The influence of the thread shape may be clearly highlighted by analyzing the stress-based performances of implants 1 and 2 and of implants 7 and 8. In particular, trapezoid-shaped thread (labelled as T10/30 in Figure 1) induced more favorable compressive and tensile states at both cortical and trabecular regions than the saw-tooth thread (T0/30), leading to the reduction of the cortical peak values of about 24% for *σ*_*C*_ when the implants D3.6-L5.5 were addressed and of about 35% for *σ*_*T*_ in the case of the implants D3.6-L9. Such an effect is also observable by analyzing the risk index *R* (see Figure 8). In particular, the thread shape T10/30 induced a significant reduction in *R* (at both cortical and trabecular regions), especially for short implants.

Finally, indications on the influence of the helical-milling width and depth may be drawn by considering numerical results relevant to implants 4 and 5 and to implants 8 and 9. Although almost comparable global stress patterns and local stress measures were experienced passing from SM (small milling) to LM (large milling), the analysis of the index *R* reveals that large milling shape can induce a reduction of the risk of overloading states at the cancellous bone, especially for small values of *L*.

### 3.2. Influence of In-Bone Positioning Depth

**Figure 9:**
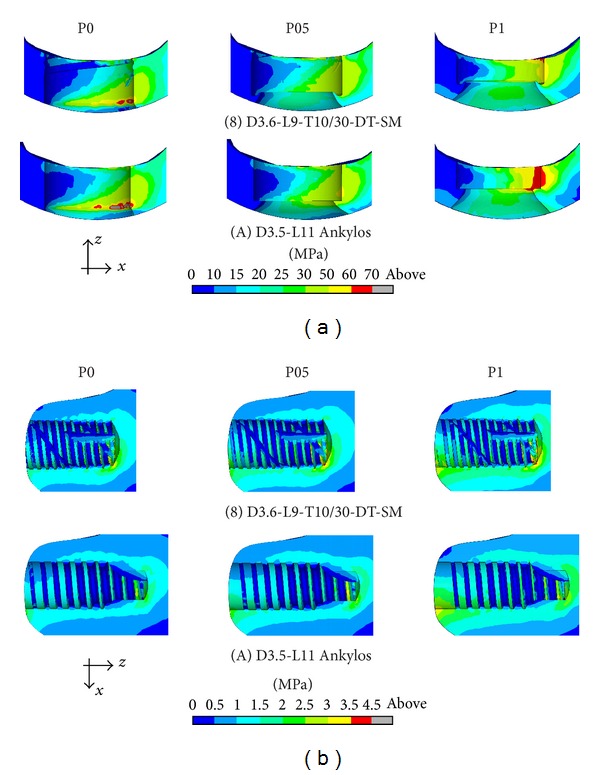
Von Mises stress contours (blue: 0; red: 70 MPa) at the coronal section *y* = 0 for implants 8 and A (see Figure 1) and for different implant in-bone positioning levels (see Figure 3). Cortical (a) and trabecular (b) peri-implant bone interface.

**Figure 10:**
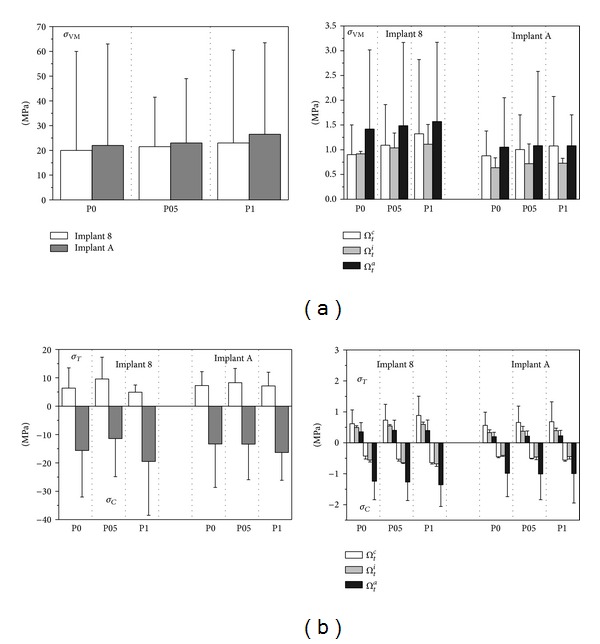
Von Mises ((a), *σ*_VM_) and principal ((b), *σ*_*T*_ tensile and *σ*_*C*_ compressive) stress measures at cortical (left side) and trabecular (right side) bone-implant interface for implants 8 and A (see Figure 1) and for different implant in-bone positioning levels (see Figure 3). Average (bars) and peak (lines) values.

**Figure 11:**
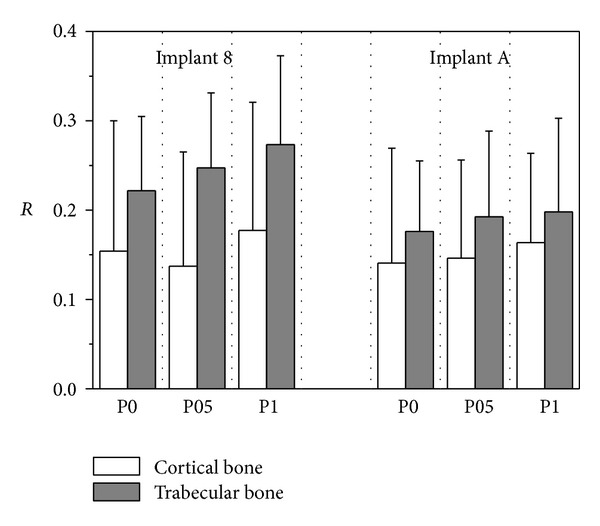
Overloading risk index *R* computed at cortical and trabecular peri-implant bone for implants 8 and A (see Figure 1) and for different implant in-bone positioning levels (see Figure 3). Average (bars) and peak (lines) values.

In order to analyze the influence of the implant in-bone positioning depth on loading transmission mechanisms, reference has been made to the comparative numerical analyses carried out for the implant D3.6-L9-T10/30-DT-SM and for the implant Ankylos D3.5-L11 (i.e., for implants 8 and A in Figure 1). Addressing the positioning configurations introduced in Figure 3, Figure 9 shows Von Mises stress distributions relevant to the loading coronal plane *y* = 0, computed at cortical and trabecular peri-implant bone regions, and Figure 10 shows mean and peak values of *σ*_VM_, *σ*_*T*_, and *σ*_*C*_ computed over the control volumes *Ω*_*c*_ and *Ω*_*t*_ (see Figure 4). Finally, Figure 11 summarizes mean and peak values of the overloading risk index *R* computed at both trabecular and cortical bone interfaces. It is worth pointing out that the results referred to the crestal positioning P0 were computed by modeling a crestal bone loss of about 10% in cortical thickness (see Figure 3).

Proposed numerical results confirmed that the implant Ankylos induced more favorable loading transmission mechanisms than implant 8, also considering different values of in-bone positioning depth. Moreover, the analysis of Von Mises stress distributions as well as of the values of principal-stress-based measures suggests that the crestal positioning (P0) induced significant stress concentrations at the cortical bone around the implant neck. In this case, stress peaks were estimated as comparable with those obtained for the subcrestal positioning P1. When the intermediate subcrestal positioning P05 was analyzed, the lowest compressive peaks at *Ω*_*c*_ were experienced for both implants, although tractions slightly greater than the other positioning configurations occurred. In trabecular bone, stress patterns were computed as almost comparable in the three cases under investigation. Nevertheless, the positioning case P0 induced stress distributions in trabecular regions that were slightly better than P05 and P1.

This evidence is fully confirmed by analyzing the results obtained for the risk index *R*. In particular, referring to its peak values, overloading risk at the cortical bone for P05 was lower than that for P0 and P1 of about 14% and 19% for implant 8, respectively, and of about 6% and 3% for implant A. On the other hand, values of *R* for P0 were lower at the trabecular bone than those for P05 and P1 of about 10% and 18% for implant 8, respectively, and of about 10% and 15% for implant A.

### 3.3. Influence of Marginal Bone Loss in Crestal Positioning

**Figure 12:**
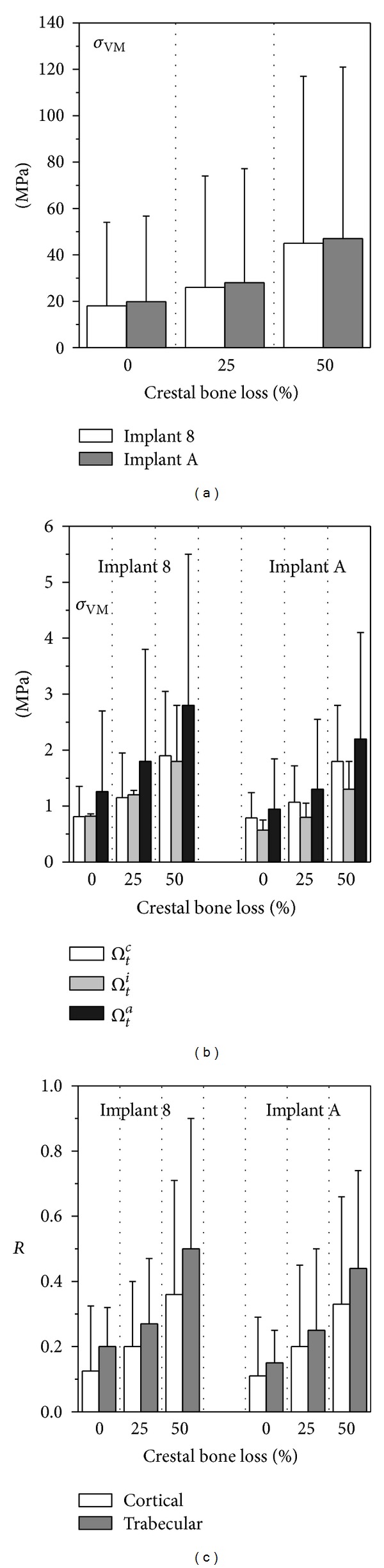
Von Mises stress measure at cortical (a) and trabecular (b) bone-implant interface for implants 8 and A (see Figure 1) and with a crestal positioning characterized by different levels of crestal bone loss. (c) Overloading risk index *R*. Average (bars) and peak (lines) values.

For implants 8 and A (see Figure 1), crestally positioned in agreement with the configuration P0 (see Figure 3), the influence of the amount in crestal bone loss was also analyzed. In particular, numerical simulations were carried out considering three different levels of marginal bone loss, from the ideal case consisting in the absence of cratering effects (bone loss equal to 0% in thickness of the cortical bone layer) up to the case of 50% bone loss. For the sake of compactness, in Figure 12 only peak and mean values of the Von Mises stress measure computed over *Ω*_*c*_ and *Ω*_*t*_ are shown, together with results computed for the overloading risk index *R*.

Numerical analyses showed that modeling an increase in cratering depth induced an increase in stress levels at both cortical and trabecular peri-implant regions and thereby induced an increase in the risk of overloading. In particular, for both implants, the Von Mises stress peaks relevant to a crestal bone loss of 50% in thickness were greater of about 120% in cortical bone and 105% in trabecular than those in the ideal case of 0% bone loss.

## 4. Discussion

The 11 dental implants that were analyzed by finite-element simulations exhibited different stress-based biomechanical behaviours, dependent on implant shape and thread, as well as on positioning depth and bone geometry around the implant neck. Simulation results considered functioning implants based on platform-switching concept and were obtained by modeling the crestal bone geometry after a healing and loading period. 

Numerical results obtained by considering a subcrestal in-bone positioning 1 mm depth of implants have highlighted the influence of implant length and diameter on load transfer mechanisms. In agreement with numerical findings obtained by other authors [37–41], an increase in implant diameter induced a significant reduction of stress peaks mainly at cortical bone, whereas the variation in implant length produced a certain influence only on stress patterns at the cancellous bone-implant interface. Accordingly, the present numerical results suggest that, in order to control overloading risk, the implant diameter can be considered as a more effective design parameter than the implant length. Similar findings were proposed in [40, 47] and were relevant also to traditional implants crestally positioned. Overloading risk, quantitatively estimated by combining compressive and tensile effects via a principal-stress-based strength criterion for bone, was computed as significant at the cortical region around the implant neck (mainly as a result of dominant compressive states induced by the lateral load component) and/or at crestal (dominant tensile states) or apical (dominant compressive states) trabecular regions (induced by the vertical intrusive load component).

Stress analyses of implants with similar length and diameter allowed us to investigate the influence of some thread features. In particular, the proposed numerical results suggest that thread shape and thread details can induce significant effects on the peri-implant stress patterns. Threads analyzed in this study were characterized by two starts and numerical results have shown that the use of the same thread truncation for both starts induced more uniform local stress distributions than the cases characterized by a different thread truncation. As regards the thread shape, trapezoid-shaped thread produced compressive and tensile states at both cortical and trabecular regions more favorable than those of the saw-tooth thread, leading to reductions in stress values that were significantly affected by implant length and diameter. Moreover, numerical evidence has highlighted that the presence of a wide helical-milling along the implant body does not significantly affect the loading transmission mechanisms, but it can contribute to reduce risks of overloading at the trabecular apical bone, especially when short implants are considered.

Numerical simulations carried out on coupled bone-implant models defined by considering different levels of the implant in-bone positioning depth have shown that a crestal placement, combined with a reduced marginal bone loss, induced great stress values at the crestal cortical regions, confirming the biomechanical relationship between the stress-based mechanical stimuli and the possible activation of bone resorption process at the implant collar [21]. In agreement with clinical evidence and with other numerical studies [4, 18, 19, 25–34, 40, 47, 53], present results confirm also that a subcrestal positioning of implants based on platform-switching concept may contribute to the preservation of the crestal bone as well as can induce more effective and homogeneous stress distributions at the peri-implant regions. In particular, proposed simulation results have shown that, in the case of subcrestal placements, stress distributions were mainly affected by two counteracting effects. On one hand, when the implant's in-bone positioning depth increases then the vertical thickness of the cortical bone engaged in load transfer mechanisms reduces, tending to generate stress concentrations. But, on the other hand, the horizontal bone apposition induced by the platform-switching configuration in a subcrestal positioning highly contributes to an effective redistribution of the stress field. As a result of a balance condition between previous effects, the best stress-based performance among cases herein analyzed has been experienced considering an in-bone positioning depth of about 25% in cortical thickness.

In the case of crestal positioning, the proposed numerical results have shown that if the crestal bone morphology, affected by possible marginal bone loss, is not properly modeled, then a significant underestimation of stress values and an inaccurate evaluation of loading transfer mechanisms are generally obtained. Moreover, the present finite-element analyses have confirmed that a progressive marginal bone loss can lead to a progressive increase in stress intensity at the peri-implant interface that, in turn, can contribute to a further overload-induced bone loss, jeopardizing clinical effectiveness and durability of the prosthetic treatment. These results are qualitatively in agreement with numerical evidence obtained in [19, 40, 41, 47] although, due to simplified and/or different models used in those studies, quantitative comparisons cannot be made.

It is worth remarking that, contrary to a number of recent numerical approaches [33, 38, 39, 41, 46], the present study accounted for the influence of posthealing crestal bone morphology in functioning implants and was based on a detailed three-dimensional geometrical modeling of the bone segment wherein the implant is inserted. Accordingly, the results herein proposed can be retained as complementary with respect to several previous simplified studies, furnishing more refined and accurate indications for choosing and/or designing threaded dental implants, as well as giving clear insights towards the understanding of main factors affecting the loading transmission mechanisms.

Although in the current study a number of aspects influencing the biomechanical interaction between dental implant and bone have been accounted for, some limitations can be found in modeling assumptions herein employed. In particular, the ideal and unrealistic condition of 100% osseous integration was assumed; stress analyses were performed by simulating static loads and disregarding any muscle-jaw interaction; bone was modeled as a dry isotropic linear elastic material, whose mechanical properties were assumed to be time independent; the space dependence of bone density and mechanical response has been simply described by distinguishing trabecular and cortical homogeneous regions. All these assumptions do not completely describe possible clinical scenarios because of possible osseointegration defects at the peri-implant regions; different patient-dependent loading distributions; much more complex and time-dependent forces and significant muscular effects; anisotropic, inhomogeneous, nonlinear, and inelastic response of living tissues; bone remodeling; and spatially graded tissue properties. Nevertheless, in agreement with other numerical studies [35–54], present assumptions can be accepted in a computational sense in order to deduce significant and clinically useful indications for the comparative stress-based assessment of threaded dental implants.

In order to enhance the present finite-element approach, future studies will be devoted to the modeling of bone as a nonlinear, anisotropic, viscous, and inhomogeneous regenerative tissue that responds to stress by resorption or regeneration under time-dependent muscular and external loads, accounting also for a more refined correlation between bone density and its mechanical response.

## 5. Concluding Remarks

Within the limitations of this study, numerical simulations showed that implant design (in terms of implant diameter, length, thread shape), in-bone positioning depth, and crestal bone morphology highly affect the mechanisms of load transmission. Aiming at the minimization of the overloading risks, the implant diameter can be retained as a more effective design parameter than the implant length. In particular, a significant reduction of stress peaks, mainly at the cortical bone, occurred when implant diameter increased. Nevertheless, implant length exhibited a certain influence on the bone-implant mechanical interaction at the cancellous interface, resulting in more effective and homogeneous stress distributions in trabecular bone when the implant length increased. Stress-based performances of dental implants were also found to be significantly affected by thread features. In detail, trapezoid-shaped thread induced compressive and tensile states at both cortical and trabecular regions more favorable than the saw-tooth thread. Moreover, the use of the same thread truncation for different thread starts induced a more uniform local stress distributions than the case of a different thread truncation. In the case of short implants, the presence of a wide helical-milling along the implant body produced a reduction in the overloading risk at the trabecular apical bone. Overloading risks were computed as high around the implant neck (for compressive states) in cortical bone and at the crestal (for tensile states) or apical (for compressive states) trabecular bone. Risk of overloading reduced when small levels of crestal bone loss were considered, as induced by suitable platform-switching strategies.
